# White Matter Microstructural Changes Using Ultra-Strong Diffusion Gradient MRI in Adult-Onset Idiopathic Focal Cervical Dystonia

**DOI:** 10.1212/WNL.0000000000209695

**Published:** 2024-08-07

**Authors:** Claire L. MacIver, Derek Jones, Katy Green, Konrad Szewczyk-Krolikowski, Andre Doring, Chantal M.W. Tax, Kathryn J. Peall

**Affiliations:** From the Cardiff University Brain Research Imaging Centre (C.L.M., D.J., K.G., A.D., C.M.W.T.), Cardiff University; Neuroscience and Mental Health Research Institute (C.L.M., K.J.P.), Division of Psychological Medicine and Clinical Neurosciences, Cardiff University School of Medicine; North Bristol NHS Trust (K.S.-K.), United Kingdom; and Image Sciences Institute (C.M.W.T.), University Medical Center Utrecht, the Netherlands.

## Abstract

**Background and Objectives:**

Adult-onset idiopathic focal cervical dystonia (AOIFCD) involves abnormal posturing of the cervical musculature and, in some individuals, an associated head tremor. Existing neuroimaging studies have implicated key motor networks. However, measures used to date lack specificity toward underlying pathophysiologic differences. We aim to assess white matter motor pathways for localized, microstructural differences, which may aid in understanding underlying mechanisms.

**Methods:**

Individuals diagnosed with AOIFCD and an age- and sex-matched control group were prospectively recruited through the Welsh Movement Disorders Research Network. All participants underwent in-depth clinical phenotyping and MRI (structural and diffusion sequences) using ultra-strong diffusion gradients. Tractography (whole-tract median values) and tractometry (along tract profiling) were performed for key white matter motor pathways assessing diffusion kurtosis imaging (DKI), neurite orientation dispersion and density imaging (NODDI), and standard model parameters. Groups were compared using linear model analysis with Bonferroni multiple comparison correction.

**Results:**

Fifty participants with AOIFCD and 30 healthy control participants were recruited, with 46 with AOIFCD and 30 healthy controls included for analysis (33 without head tremor, 13 with head tremor). Significant differences were observed in the anterior thalamic radiations (lower mid-tract fractional anisotropy [estimate = −0.046, *p* = 3.07 × 10^−3^], radial kurtosis [estimate = −0.165, *p* = 1.42 × 10^−4^], *f*–intra-axonal signal fraction [estimate = −0.044, *p* = 2.78 × 10^−3^], *p*_2_ orientation coherence [estimate = −0.043, *p* = 1.64 × 10^−3^], higher Orientation Dispersion Index [ODI, estimate = 0.023, *p* = 2.22 × 10^−3^]) and thalamopremotor tracts (higher mid-tract mean kurtosis [estimate = 0.064, *p* = 7.56 × 10^−4^], lower Neurite Density Index [estimate = 0.062, *p* = 2.1 × 10^−3^], higher distal tract ODI [estimate = 0.062, *p* = 3.1 × 10^−3^], lower *f* [estimate = −0.1, *p* = 2.3 × 10^−3^], and striatopremotor tracts [proximal lower *f*: estimate = −0.075, *p* = 1.06 × 10^−3^]). These measures correlated with clinical measures: dystonia duration (right thalamopremotor distal ODI: *r* = −0.9, *p* = 1.29 × 10^−14^), psychiatric symptoms (obsessive compulsive symptoms: left anterior thalamic radiation *p*_2_ *r* = 0.92, *p* = 2.797 × 10^−11^), sleep quality (Sleep Disorders Questionnaire Score: left anterior thalamic radiation ODI: *r* = −0.84, *p* = 4.84 × 10^−11^), pain (left anterior thalamic radiation ODI: *r* = −0.89, *p* = 1.4 × 10^−13^), and cognitive functioning (paired associated learning task *p*_2_, *r* = 0.94, *p* = 6.68 × 10^−20^).

**Discussion:**

Overall, localized microstructural differences were identified within tracts linking the prefrontal and premotor cortices with thalamic and basal ganglia regions, suggesting pathophysiologic processes involve microstructural aberrances of motor system modulatory pathways, particularly involving intra-axonal and fiber orientation dispersion measures.

## Introduction

Adult-onset idiopathic focal cervical dystonia (AOIFCD) is the most common form of adult-onset idiopathic dystonia. Cervical muscle overactivity and loss of coordinated muscle contraction lead to abnormal posturing and pain, with a proportion of patients experiencing an associated head tremor. Pathophysiologic understanding of AOIFCD is limited, resulting in a substantial unmet health need, with current treatment options comprising symptomatic rather than disease-modifying therapies, often having incomplete efficacy and significant remaining symptom burden. Findings from human functional brain imaging studies of focal dystonia indicate reduced motor network–based functional connectivity^1,2^ and disruption to inhibitory/excitatory neurotransmitter balance, including reduced thalamic GABA levels.^3^ With respect to structural imaging, gray matter volumetric differences have been most consistently demonstrated among task-specific dystonias with increased volumes in motor gray matter^4^ and more variable findings observed in AOIFCD cohorts.^5,6^ White matter analysis in AOIFCD has predominantly involved diffusion MRI, using relatively nonspecific measures, coupled with heterogeneous approaches and findings. Some have demonstrated localized higher, lower, or unchanged diffusion tensor measures such as fractional anisotropy (FA) and mean diffusivity (MD) within motor pathways.^5,7-10^

By contrast, monogenic forms of dystonia have demonstrated more consistent findings, including lower FA in motor pathways connecting the cerebellum, basal ganglia structures, and cortical motor gray matter, coupled with indications of more widespread white matter pathology.^11-14^ More recently, tremor-specific imaging differences have been suggested, with higher cerebellar peduncular FA observed in dystonic tremor compared with other tremor syndromes.^8,9^ These differences between disparate genotypes and phenotypes of dystonia may suggest that interpretations, such as lower white matter tract integrity, are not a unifying feature across the spectrum of dystonic disorders. In addition, parameters such as FA based on diffusion tensor MRI, comprising most of the existing literature, are of a nonspecific nature and have the potential to represent a range of underlying biological changes.^15^

Enhancing our understanding of the neuroanatomy of motor control networks in AOIFCD is a vital step in progressing diagnostic and therapeutic management strategies for this patient group. To date, few attempts have been undertaken to assess more microstructurally relevant parameters, including using modeling and other approaches that compartmentalize the intra and extra-axonal signal with the aim to more specifically quantify tissue characteristics. One fixel-based analysis approach has described lower apparent fiber density localized in the region of the striatum^16^ in AOIFCD, and one microstructural modeling-based study identified localized regions of both higher and lower orientation dispersion using neurite orientation dispersion and density imaging (NODDI) in the superior cerebellar peduncles and anterior thalamic radiations in a mixed dystonia cohort.^9^ This later study found no differences with whole-tract averaging (tractography), whereas profiling of changes to parameters along the tracts (tractometry) identified localized differences. These approaches, however, have been undertaken using acquisitions that consist of only low-to-moderate *b* values (up to *b* = 2,000 s/mm^2^), with potential for inaccuracy in modeled parameters stemming from the a priori fixing of values to address model degeneracy.^17^ To overcome some of these limitations, diffusion MRI approaches that make use of more advanced acquisitions, such as multiple *b*-value measurements that include higher *b*-values (>2,000 s/mm^2^) or multiple *b*-tensor encoding schemes,^18^ have been exploited to optimize modeling approaches and negate the need for extensive a priori fixing of parameters.^19^

We use ultra-strong diffusion gradient MRI scanning in a deeply phenotyped AOIFCD cohort to explore white matter microstructural differences in pathways linking key motor brain hubs and, if present, anticipate accentuation of these differences through application of optimal acquisition and microstructural modeling approaches.

## Methods

### Standard Protocol Approvals, Registrations, and Participant Consents

Participant recruitment was done using the Welsh Movement Disorders Research Network (REC reference: 14/WA/0017 IRAS ID: 146495), with clinical phenotyping performed under the Global Myoclonus Dystonia Registry and Non-Motor Symptoms Study (REC reference: 18/WM/0031, IRAS project ID: 236219). Ethical approval for brain imaging was obtained from the Cardiff University School of Medicine Research Ethics Committee (SMREC reference number 18/30). Written informed consent was obtained for all participants in the study.

### Recruitment

Study inclusion required a clinical diagnosis of AOIFCD, diagnosed by a neurologist with expertise in movement disorders, with exclusion criteria including existence of a contraindication to MR scanning, significant claustrophobia, inability to tolerate the scan, coexistence of other neurologic diagnoses, or a diagnosis of another form of dystonia. Recruited individuals with AOIFCD underwent whole-exome sequencing genetic analysis for evidence of pathogenic variants in known dystonia-causing genes. Those identified with genetic variants were not included in this study.

### Clinical Characterization

During the assessment visit on the day of the scan, a videotaped clinical examination was undertaken following a modified form of the Burke-Fahn-Marsden Dystonia Scale (BFMDRS) protocol that would also allow for scoring with both the BFMDRS and Toronto West Spasmodic Torticollis Rating Scale (TWSTRS). These were reviewed and scored independently by 2 neurologists with expertise in movement disorders.

Extensive non-motor phenotypic data were additionally acquired. The participants undertook a self-completed questionnaire assessing basic demographic information, psychiatric symptomatology (the modified Mini International Neuropsychiatric Interview Score, Structured Clinical Interview for DSM-5 Personality Disorders, Beck Depression inventory, Health Anxiety Index, and Yale-Brown Obsessive Compulsive scale), sleep (Pittsburgh Sleep Quality Index,^20^ Sleep Disorders Questionnaire,^21^ and Epworth Sleepiness Scale), pain (typical pain time course, Chronic Pain Acceptance Questionnaire, Pain Catastrophizing Scale), and quality of life (Short Form 36 Health survey [SF36]). Cognitive function was assessed face-to-face with the National Adult Reading Test used to determine IQ and the Cambridge Neuropsychological Test Automated Battery (CANTAB^22^) used to assess multiple cognitive domains (One Touch Stockings of Cambridge [executive function], emotional recognition task, paired associated learning, and spatial working memory).

### MRI Data Acquisition

#### Scanning Acquisition

All imaging were undertaken on a 3T Connectom scanner (Siemens Healthcare), with a 32-channel head coil and a scan time for white matter assessment of approximately 45 minutes. An initial structural scan was undertaken using a sagittal T1-weighted magnetization prepared rapid gradient echo imaging acquisition, with the acquisition using a repetition time of 2,300 milliseconds, echo time 2 milliseconds, flip angle 9°, and inversion time 857 milliseconds. The field of view was 192 × 256 × 256 mm, with a voxel size of 1 × 1 × 1 mm. Diffusion MRI data were acquired for the whole brain with multiple *b*-values (*b* = 0 s/mm^2^ [19 directions], *b* = 200 s/mm^2^ [20 directions], *b* = 500 s/mm^2^ [20 directions], *b* = 1,200 s/mm^2^ [30 directions], *b* = 2,400 s/mm^2^ [61 directions], *b* = 4,000 s/mm^2^ [61 directions], *b* = 6,000 s/mm^2^ [61 directions], *b* = 30,000 s/mm^2^ [120 directions]). Gradient sampling vectors were distributed across the entire unit sphere. The acquisition used a repetition time of 4,000 milliseconds and echo time of 75 milliseconds, Δ 30 milliseconds and δ 15 milliseconds, and flip angle 90°. The field of view was 110 × 110 × 66 mm, with a voxel size of 2 × 2 × 2 mm. No partial Fourier was used. An additional *b* = 0 image was acquired with a reversed phase encoding direction.

#### Preprocessing

**Figure 1:**
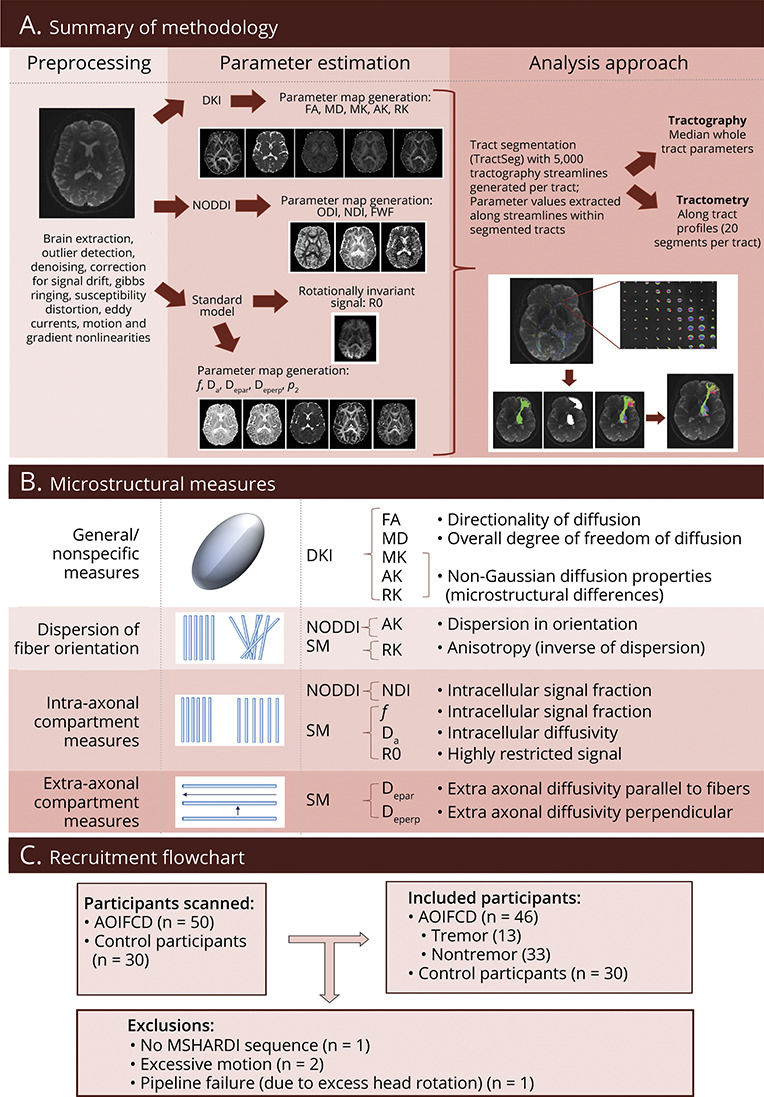
Schematic of Methodological Approaches and Recruitment (A) Overview of analysis methodology. (B) Schematic of measured parameters. (C) Recruitment flowchart. AK = axial kurtosis; D_a_ = intra-axonal diffusivity; D_epar_ = extra-axonal parallel diffusivity; D_eperp_ = extra-axonal perpendicular diffusivity; DKI = diffusion kurtosis imaging; *f* = intraneurite signal fraction; FA = fractional anisotropy; FWF = free water fraction; MD = mean diffusivity; MK = mean kurtosis; NDI = Neurite Density Index; NODDI = neurite orientation dispersion and density index; ODI = Orientation Distribution Index; *p*_2_ = orientation coherence; R0 = rotationally invariant signal for order = 0, *b* = 6,000 s/mm^2^; RK = radial kurtosis; SM = standard model.

The overall preprocessing and analysis approach is presented in Figure 1A. The T1-weighted acquisition underwent on-scanner distortion correction while the diffusion acquisition underwent brain extraction; denoising (Marchenko-Pastur principal component analysis–based approach)^23^; outlier detection (e.g., because of signal dropout from motion)^24^; signal drift correction; and correction for Gibbs ringing,^25^ eddy currents, susceptibility distortion and subject motion (using FMRIB Software Library [FSL, version 6.0.5] eddy),^26^ and gradient nonlinearities.^27,28^ The FSL eddy step included “repol” (where outlier slices were removed and replaced using estimates) and “slice-to-vol” motion correction for the participants with significant between-slice motion (where individual slices moved by motion were realigned to the overall volume).

#### Diffusion Parameter Estimates

1. Diffusion tensor imaging (DTI) and diffusion kurtosis imaging (DKI): Diffusion tensor (FA, MD) and diffusion kurtosis (MK-mean kurtosis, AK-axial kurtosis, RK-radial kurtosis) parameters were estimated using a constrained weighted least squares fitting approach with weight reduction for outlier measurement.^28,29^
2. Microstructural modeling: (a) NODDI: estimation of parameter maps for Orientation Dispersion Index (ODI), Neurite Density Index (NDI), and free water fraction (FWF)^30^; (b) standard model (SM) of white matter: a more general white matter model was fit using fewer constraints. This approach involved a least squares estimation of rotationally invariant spherical harmonics of the diffusion signal up to L = 4, using the acquired b-shells up to *b* = 6,000 s/mm^2^, with Rician bias correction included. From this, the SM parameters were estimated with data-driven machine learning regression,^31^ applying a 3-compartment model (intra-axonal signal, extra axonal signal, and free water). Parameter maps were estimated for *f* (intra-axonal signal fraction), D_a_ (intra-axonal diffusivity), D_epar_ (extra-axonal parallel diffusivity) and D_eperp_ (extra-axonal perpendicular diffusivity), and *p*_2_ (measure for orientation coherence). In addition to the modeled parameters, the raw rotationally invariant signal for L = 0, *b* = 6,000 s/mm^2^ (R0), was assessed without addition of constraints or model fitting that was considered reflective of intra-axonal signal.^32^

Across the modeling approaches, parameters aimed to represent orientational dispersion of axon bundles (NODDI: ODI—the degree of dispersion of axons; SM: *p*_2_—the degree of orientation coherence, therefore inversely related to the ODI); measures relating to aspects of intra-axonal signal generally taken to be reflective of axonal density (NODDI: NDI—the proportion of signal attributable to the intra-axonal compartment; SM: *f*—the proportion of signal attributable to the intra-axonal compartment; D_a_—the degree of diffusivity in the intra-axonal compartment; R0—rotationally invariant spherical harmonics of the signal (at L = 0, *b* = 6,000) hypothesized to correspond to intra-axonal signal); and measures relating to the extra-axonal compartment (SM: D_epar_—the degree of diffusivity in the extra-axonal space parallel to the axonal compartment, D_eperp_—the degree of diffusivity in the extra-axonal space perpendicular to the axonal compartment) (Figure 1B).

#### Tract Segmentation

White matter tracts of interest were segmented using Tractseg,^33^ replicating our previous approach in an undifferentiated dystonia cohort^9^: middle cerebellar peduncle, bilateral inferior cerebellar peduncles, superior cerebellar peduncles, frontopontine tracts, corticospinal tracts, anterior thalamic radiations, superior thalamic radiations, thalamoprefrontal tracts, thalamopremotor tracts, thalamoprecentral tracts, thalamopostcentral tracts, striatopremotor tracts, striatoprecentral tracts, striatopostcentral tracts, and optic radiations (used as a non-motor comparator tract). Multishell multitissue constrained spherical deconvolution was applied to the preprocessed diffusion data, with peak extraction (maximum of 3 peaks per voxel). A fully connected convolutional neural network then created a tract probability image for each orientation and tract. Start and end regions were segmented, with fiber orientation maps calculated within each segmented tract.^34^

### Analysis

Probabilistic tractography^35^ with 5,000 streamlines was performed for each tract. Whole-tract median values for each parameter were extracted within segmented tracts for the tractography analysis. Given the implication from previous work that white matter differences are localized rather than tract-wide,^9^ tractometry analysis was performed for each parameter,^36,37^ involving assessing the profile of values and how they change across the length of the tract. For the tractometry analysis, tracts were split lengthwise into 20 equal segments, identifying the centroid of all streamlines within each segment. The end 2 segments were excluded, with the median parameter value across streamlines calculated for each of the 18 remaining segments.^37^ Tractometry was not performed for the thalamoprefrontal, thalamoprecentral, striatoprecentral, thalamopostcentral, or striatopostcentral tracts as their geometry does not allow for consistent segmentation.^38^

### Excluded Data

After MR scanning, participants were excluded if there was significant motion artifact evident on the preprocessed diffusion acquisitions, assessed using FSL EddyQC and by manual inspection.

### Statistical Analysis

Analysis was undertaken using RStudio version 0.99.892. Participant demographic and clinical phenotypic data were summarized using appropriate descriptive statistics dependent on data distribution. Regression models were applied comparing disease status (independent variable) for each parameter (dependent variable), with age, sex, and handedness included as covariates. Additional analyses were performed for those with and without associated tremor, with each group being compared with the control cohort. Bonferroni correction for multiple comparisons was undertaken for parameters, with differences reported only if significant after correction for multiple comparisons, unless otherwise stated. Correlation analyses were used to examine for association between the clinical phenotypic traits and the significant diffusion parameters using Pearson correlation coefficients.

### Data Availability

Anonymized data will be made available on reasonable request.

## Results

Fifty individuals diagnosed with AOIFCD and 30 age and sex-matched control participants were recruited. Of these, 4 participants from the AOIFCD cohort were excluded because of incomplete imaging acquisition (n = 1), excess movement artifact (n = 2), and failure of the analytical pipeline (n = 1) (Figure 1C). The participants were matched for age at examination, with median age of the AOIFCD cohort 55.5 years (range 30–74 years) and 57 years (range 32–72 years) for the control cohort (*p* = 0.919), and sex, with a male:female ratio of 12:34 in the AOIFCD cohort and 9:21 in the control cohort (*p* = 0.912). The median age at onset of dystonia was 43.5 years (range 30–67 years) and median time since dystonia diagnosis 12.5 years (range 1–33 years). Demographic characteristics are summarized in Table 1, with no significant differences observed between cohorts, except for a lower median IQ score within the tremor cohort (median 111.4) compared with controls (118.9) (*p* = 8.14 × 10^−3^), which while statistically significant is a small difference in terms of clinical IQ measurements. An overview of the full motor and non-motor phenotypic characteristics of the cohort is summarized in eTables 1 and 2. A significantly higher burden of these symptoms was observed across all non-motor domains in the AOIFCD cohort, including overall psychiatric symptomatology (modified MINI total score—AOIFCD: median 4, range 0–15; controls: median 1, range 0–9; *p* = 2.49 × 10^−4^, depression Beck Depression Inventory—AOIFCD: median 11, range 0–38; controls: median 8, range 1–38; *p* = 0.0199), sleep disturbance (Pittsburgh Sleep Quality Index—AOIFCD: median 9, range 2–20; controls: median 6.5, range 2–15, *p* = 0.004; Sleep Disorders Questionnaire—AOIFCD: median 18, range 7–50; controls: median 9, range 6–26; *p* = 5.78 × 10^−5^), pain (Pain Catastrophizing Scale—AOIFCD: median 12, range 0–47; controls: median 5, range 0–22; *p* = 0.004), spatial working memory (total errors on task—AOIFCD: median 16.5, range 0–36; controls: median 6.5, range 0–36; *p* = 0.003), and overall quality of life (physical functioning–AOIFCD: median 60, range 5–100; controls: median 90, range 35–100; *p* = 2.31 × 10^−4^).

**Table:** Summary of Clinical Characteristics of Participants Across Cohorts

Characteristic	Healthy controls Median (range)	Overall AOIFCD		AOIFCD without tremor		AOIFCD with tremor	
		Median (range)	*p* Value	Median (range)	*p* Value	Median (range)	*p* Value
No. of participants	30	46	—	33	—	13	—
Age at examination, y, median (range)	57 (32–72)	55.5 (30–74)	0.919	55 (30–74)	0.911	56 (40–69)	0.966
Sex (male:female)	9:21	12:34	0.912	8:25	0.818	4:9	1
Handedness (right:left)	29:1	43:3	0.934	31:2	1	12:1	1
IQ (NART), median (range)	118.9 (102.3–125.5)	115.5 (100.6–125.5)	0.075	117.2 (100.6–125.5)	0.433	111.4 (101.4–122.2)	8.14 × 10^−3a^
Age of dystonia onset, y, median (range)	—	43.5 (20–67)	—	44 (20–67)	—	41.5 (30–62)	—
Duration of dystonia, median (range)	—	12.5 (1–33)	—	12.5 (1–33)	—	12.5 (3–29)	—
Smoker, n (%)	3 (10)	8 (17)	0.594	5 (15)	0.366	3 (23)	0.234
BFMDRS							
Rater 1, median (range)	—	7 (1.5–21)	—	—	—	—	—
Rater 2, median (range)	—	7.5 (1.5–18)	—	—	—	—	—
Inter-rater correlation coefficient	—	0.81	—	—	—	—	—
TWSTRS							
Rater 1, median (range)	—	17 (5–23)	—	—	—	—	—
Rater 2, median (range)	—	19 (5–23)	—	—	—	—	—
Inter-rater correlation coefficient	—	0.66	—	—	—	—	—
Head tremor present (yes:no)	—	33:13	—	—	—	—	—
Dystonia medication, n (%)							
Botulinum toxin	—	37 (80)	—	25 (76)	—	12 (92)	—
Propranolol	—	1 (2)	—	1 (3)	—	0 (0)	—
Trihexyphenidyl	—	4 (8)	—	4 (12)	—	0 (0)	—
Amitryptiline/nortryptiline	—	5 (10)	—	3 (9)	—	2 (15)	—
Prebabalin/gabapentin	—	4 (8)	—	2 (6)	—	2 (15)	—
Baclofen	—	2 (4)	—	2 (6)	—	0 (0)	—
Diazepam	—	3 (6)	—	3 (9)	—	0 (0)	—
Simple analgesics	—	8 (17)	—	5 (15)	—	3 (23)	—
Clonazepam	—	3 (6)	—	2 (6)	—	1 (8)	—
Duloxetine	—	1 (2)	—	0 (0)	—	1 (8)	—
Co-careldopa	—	1 (2)	—	1 (3)	—	0 (0)	—

Abbreviations: NART = National Adult Reading Test; BFMDRS = Burke-Fahn-Marsden Dystonia Rating Scale; TWSTR = Toronto West Spasmodic Torticollis Rating Scale.

a Significant difference.

### Tractography

Tractography analysis demonstrated no significant differences in median values in the overall AOIFCD cohort, tremor or non-tremor subgroups, compared with controls for any of the measured parameters including DTI/DKI, NODDI, SM, or rotationally invariant spherical harmonics (eFigures 1–3).

### Tractometry

#### Overall Dystonia Cohorts vs Controls

**Figure 2:**
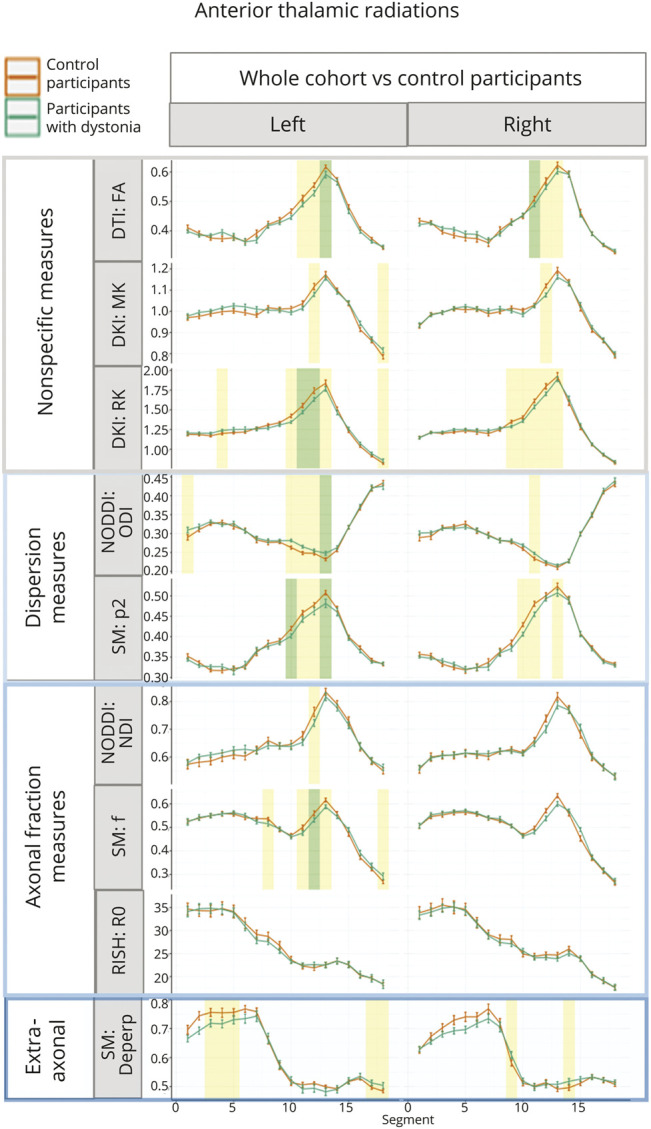
Along-Tract Profiles for the Anterior Thalamic Radiations Parameters are shown only where significant differences were demonstrated in any analyses, with median tract value and standard error displayed for each tract. Green-shaded regions represent those significantly different after multiple comparison correction, and yellow-shaded regions are significant before correction. D_eperp_ = extra-axonal perpendicular diffusivity; DKI = diffusion kurtosis imaging; DTI = diffusion tensor imaging; *f* = intraneurite signal fraction; FA = fractional anisotropy; MK = mean kurtosis; NDI = Neurite Density Index; NODDI = neurite orientation dispersion and density imaging; ODI = Orientation Dispersion Index; *p*_2_ = orientational coherence; R0 = rotationally invariant signal for order = 0, *b* = 6,000 s/mm^2^; RISH = rotationally invariant spherical harmonics; RK = radial kurtosis; SM = standard model.

**Figure 3:**
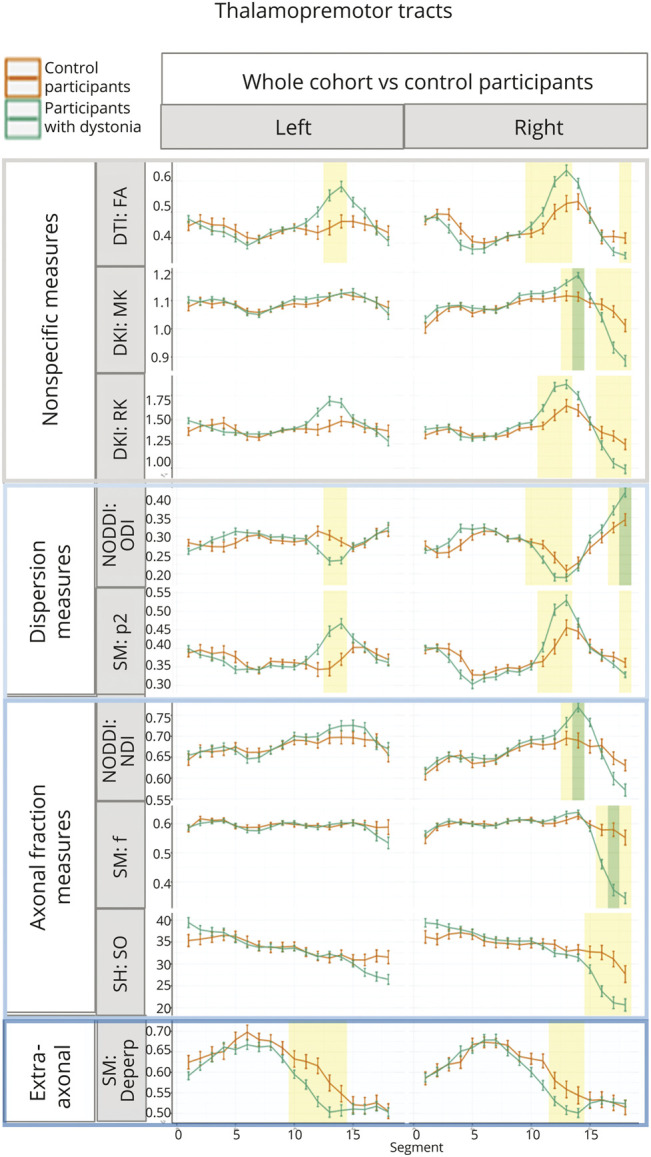
Along-Tract Profiles for the Thalamopremotor Tracts Parameters are shown only where significant differences were demonstrated in any analyses, with median tract value and standard error displayed for each tract. Green-shaded regions represent those significantly different after multiple comparison correction, and yellow-shaded regions are significant before correction. FA = fractional anisotropy; DKI = diffusion kurtosis imaging; DTI = diffusion tensor imaging; MK = mean kurtosis; RK = radial kurtosis; NODDI = neurite orientation dispersion and density imaging; SM = standard model; ODI = Orientation Dispersion Index; *p*_2_ = orientational coherence; NDI = Neurite Density Index; *f* = intraneurite signal fraction; RISH = rotationally invariant spherical harmonics; R0 = rotationally invariant signal for order = 0, *b* = 6,000 s/mm^2^; D_eperp_ = extra-axonal perpendicular diffusivity.

**Figure 4:**
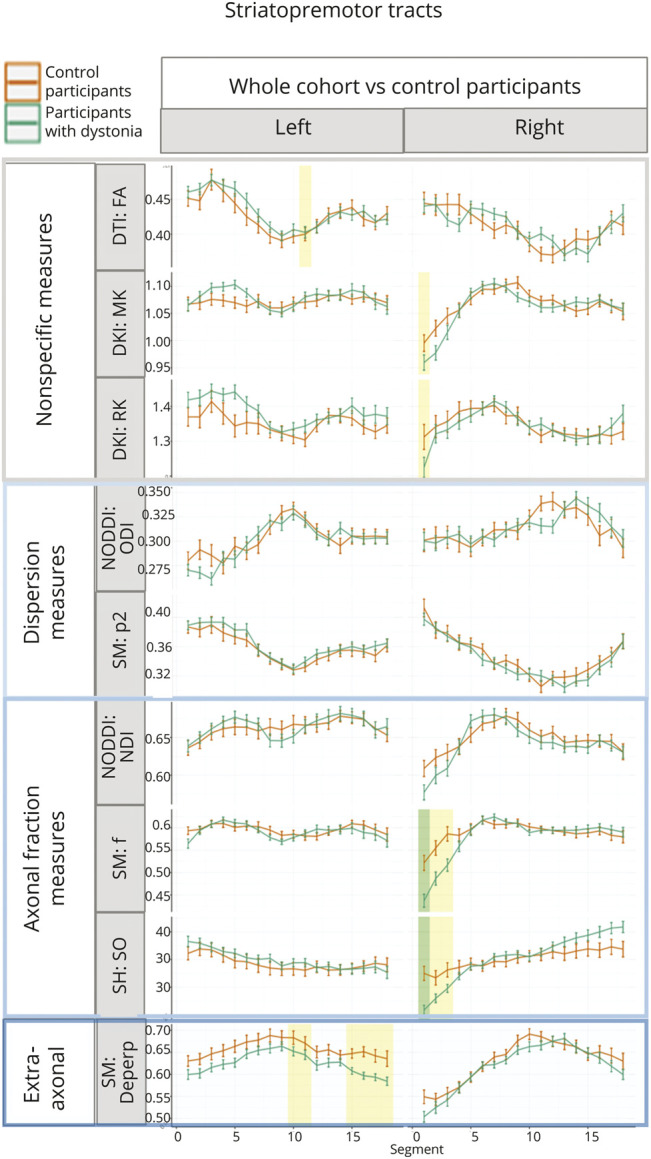
Along-Tract Profiles for the Striatopremotor Tracts Parameters are shown only where significant differences were demonstrated in any analyses, with median tract value and standard error displayed for each tract. Green-shaded regions represent those significantly different after multiple comparison correction, and yellow-shaded regions are significant before correction. FA = fractional anisotropy; DKI = diffusion kurtosis imaging; DTI = diffusion tensor imaging; MK = mean kurtosis; RK = radial kurtosis; NODDI = neurite orientation dispersion and density imaging; SM = standard model; ODI = Orientation Dispersion Index; *p*_2_ = orientational coherence; NDI = Neurite Density Index; *f* = intraneurite signal fraction; RISH = rotationally invariant spherical harmonics; R0 = rotationally invariant signal for order = 0, *b* = 6,000 s/mm^2^; D_eperp_ = extra-axonal perpendicular diffusivity.

Localized significant differences were observed in anterior thalamic radiations (Figure 2), thalamopremotor tracts (Figure 3), and striatopremotor tracts (Figure 4; eFigures 4–6). In the anterior thalamic radiations, FA values were significantly lower on both right (estimate = −0.031, *p* = 2.68 × 10^−3^) and left (estimate = −0.046, *p* = 3.07 × 10^−3^) sides coupled with corresponding lower RK (estimate = −0.165, *p* = 1.42 × 10^−4^) and higher ODI NODDI values (estimate = 0.023, *p* = 2.22 × 10^−3^) on the left. The SM analysis showed lower associated *p*_2_ (estimate = −0.043, *p* = 1.64 × 10^−3^) and *f* (estimate = −0.044, *p* = 2.78 × 10^−3^), again on the left side. The thalamopremotor tracts revealed right-sided mid and distal tract differences, with higher mid-tract MK (estimate = 0.064, *p* = 7.56 × 10^−4^) and associated lower NDI (estimate = 0.062, *p* = 2.1 × 10^−3^), with distal tract higher ODI (estimate = 0.062, *p* = 3.1 × 10^−3^) and lower *f* (estimate = −0.1, *p* = 2.3 × 10^−3^). Changes in striatopremotor tract were observed in the proximal portion of the right tract with lower *f* (estimate = −0.075, *p* = 1.06 × 10^−3^) (SM) and corresponding lower R0 (estimate = −4.572, *p* = 2 × 10^−3^) (unconstrained rotational invariant signal measure).

#### AOIFCD Without Head Tremor vs Controls

Statistically significant differences were observed in the left anterior thalamic radiations (eFigure 7), right-sided thalamopremotor tract (eFigure 8), and bilateral sided striatopremotor tract (eFigure 9). The left anterior thalamic radiation showed differences with lower FA (estimate = −0.05, *p* = 1.32 × 10^−3^), lower RK (estimate = −0.168, *p* = 4.54 × 10^−4^), and higher ODI (estimate = 0.028, *p* = 2.93 × 10^−4^), coupled with corresponding lower *p*_2_ (estimate = −0.051, *p* = 6.43 × 10^−4^) and *f* (estimate = −0.05, *p* = 1.63 × 10^−3^) (SM) in the dystonia cohort compared with controls. In the right-sided thalamopremotor tract, higher distal tract ODI (estimate = 0.071, *p* = 8.87 × 10^−4^), lower *f* (estimate = −0.099, *p* = 3.54 × 10^−3^), lower R0 (estimate = −7.241, *p* = 2.26 × 10^−3^), mid-tract higher MK (estimate = 0.066, *p* = 1.35 × 10^−3^), and lower NDI (estimate = 0.07, *p* = 1.65 × 10^−3^) were observed in the dystonia cohort compared with controls. In the proximal right-sided striatopremotor tract, lower R0 (estimate = −4.9, *p* = 2.94 × 10^−3^) was observed, with lower mid-tract D_eperp_ (estimate = −0.055, *p* = 3.46 × 10^−3^) on the left side. In the optic tract, the only difference was seen on the right side only in a single measure (higher ODI, estimate = 0.027, *p* = 1.89 × 10^−3^) and a single segment (eFigure 4).

#### AOIFCD With Head Tremor vs Controls

Significant differences were only observed with NODDI analysis of the frontopontine tract (eFigure 4), with 2 regions of lower ODI compared with controls (estimate = −0.016, *p* = 2.4 × 10^−3^ and estimate = −0.037, *p* = 2.57 × 10^−3^).

#### Correlation of Clinical and Imaging Parameters

**Figure 5:**
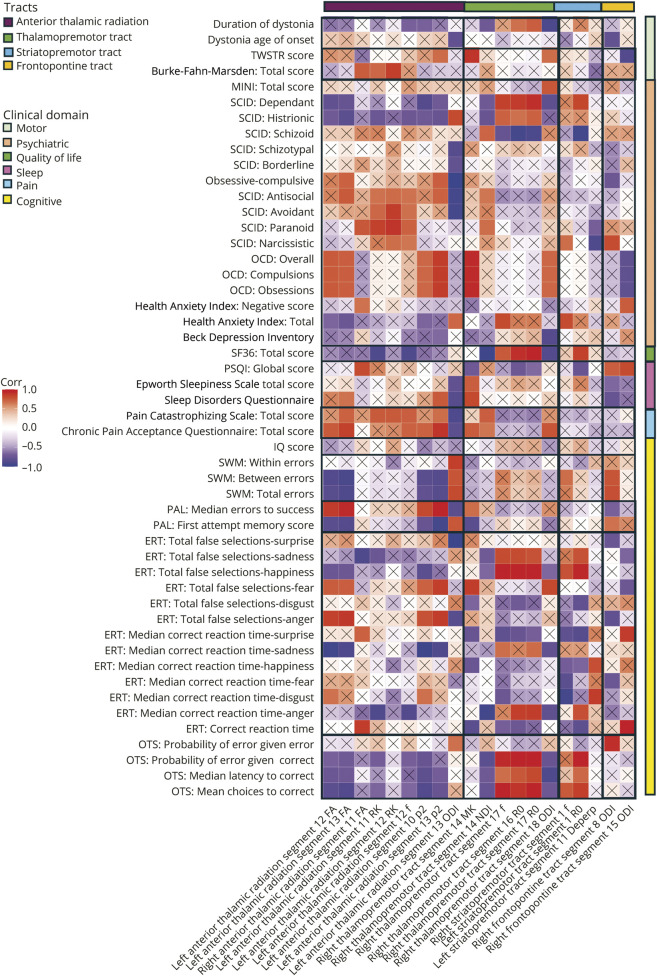
Summary of Pearson Correlation Analyses Within the Dystonia Cohort for Clinical Characteristics and Imaging Features That Were Significantly Different in the Dystonia Cohort Compared With Controls Correlations marked with x were not statistically significant after correction for multiple comparisons. TWSTR = Toronto West Spasmodic Torticollis Rating Scale; MINI = modified Mini International Neuropsychiatric Interview; SCID = Structured Clinical Interview for DSM-V Personality Disorders Questionnaire; OCD = Yale-Brown Obsessive-Compulsive Scale; SF36 = Short Form 36 Health Survey; PSQI = Pittsburgh Sleep Quality Index; SWM = spatial working memory; PAL = paired associated learning; ERT = emotional recognition task; OTS = One Touch Stockings of Cambridge; FA = fractional anisotropy; MK = mean kurtosis; RK = radial kurtosis; ODI = Orientation Dispersion Index; *p*_2_ = orientational coherence; NDI = Neurite Density Index; *f* = intraneurite signal fraction; R0 = rotationally invariant signal for L = 0, *b* = 6,000 s/mm^2^; D_eperp_ = extra-axonal perpendicular diffusivity.

Figure 5 shows the correlation plots for the clinical parameters compared with the significant imaging feature, with extensive correlations of measures across domains. The left anterior thalamic radiation mid-tract RK correlated with Burke-Fahn-Marsden score (*r* = 0.85, *p* = 4.3 × 10^−11^). ODI negatively correlated with SCID personality traits including obsessive-compulsive traits (*r* = −0.97, *p* = 1.14 × 10^−26^), and *p*_2_ positively correlated with OCD scores (*r* = 0.92, *p* = 2.797 × 10^−11^). The SF36 quality-of-life questionnaire showed anterior thalamic radiation correlations across metrics, the strongest as a negative correlation with mid tract (*f*, *r* = −0.88, *p* = 4.142 × 10^−13^). Sleep symptoms correlated with ODI (Sleep Disorders Questionnaire total score: *r* = −0.84, *p* = 4.84 × 10^−11^). Widespread tract correlations were seen with pain scores, with the strongest a negative correlation with ODI (*r* = −0.89, *p* = 1.4 × 10^−13^). In the CANTAB cognitive testing, correlations were seen across measures in the spatial working memory task (mid-tract FA, *r* = −0.91, *p* = 4.58 × 10^−15^), paired associated learning task (mid tract *p*_2_, *r* = 0.94, *p* = 6.68 × 10^−20^), and emotional recognition task (sadness; mid tract *p*_2_, *r* = −0.96, *p* = 6.99 × 10^−13^).

The right thalamopremotor tract distal changes were negatively correlated with duration of dystonia (ODI, *r* = −0.9, *p* = 1.29 × 10^−14^) and mid-tract changes positively correlated with the TWSTR score (MK, *r* = 0.9, *p* = 2.94 × 10^−14^). There was correlation with schizoid (*r* = −0.93, *p* = 3.97 × 10^−17^) and dependent (*r* = 0.91, *p* = 8.29 × 10^−16^) personality traits, quality of life (mid-tract negative correlation with NDI, *r* = −0.96, *p* = 4.03 × 10^−23^, distal tract positive correlation with R0, *r* = 0.94, *p* = 3.27 × 10^−19^), and sleep (Sleep Disorders Questionnaire, MK *r* = 0.82, *p* = 2.79 × 10^−9^). In the CANTAB cognitive testing, correlations were seen particularly in the emotional recognition task (happiness; *f*, *r* = 0.95, *p* = 2.12 × 10^−21^) and executive functioning task (distal thalamopremotor tract R0, *r* = 0.99, *p* = 5.34 × 10^−42^).

The striatopremotor tract measures included correlations in domains including personality traits (schizoid, R0: *r* = −0.94, *p* = 1.03 × 10^−18^) and quality of life (SF36, R0, *r* = 0.84, *p* = 2.69 × 10^−10^). Cognitive domains were additionally associated, including in the emotional recognition task (happiness, *r* = 0.93, *p* = 5.54 × 10^−18^) and executive functioning task (*r* = 0.95, *p* = 4.97 × 10^−20^). The frontopontine tract measures correlated with TWSTR (*r* = −0.87, *p* = 4.39 × 10^−12^), obsessive-compulsive symptoms (*r* = 0.8, *p* = 1.7 × 10^−8^), sleep quality (*r* = −0.81, *p* = 1.08 × 10^−8^), and emotional recognition task (*r* = 0.96, *p* = 5.52 × 10^−23^).

## Discussion

**Figure 6:**
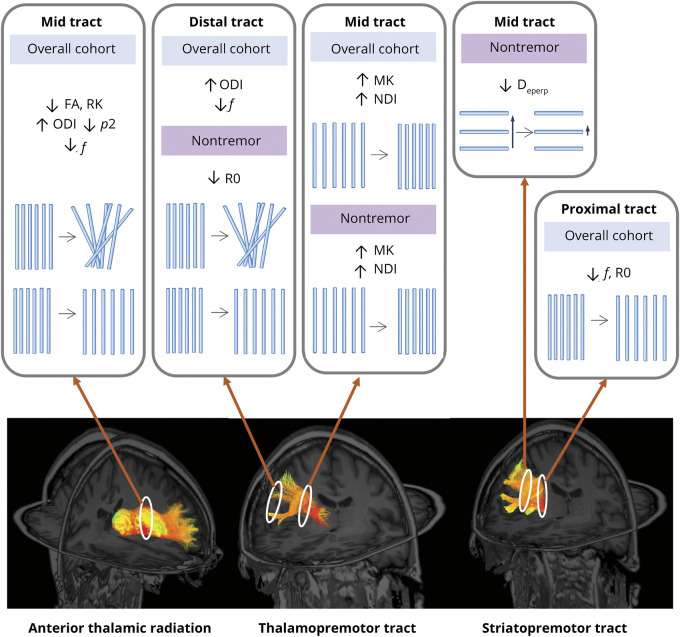
Summary of Key Findings FA = fractional anisotropy; MK = mean kurtosis; RK = radial kurtosis; ODI = Orientation Dispersion Index; *p*_2_ = orientational coherence; NDI = Neurite Density Index; *f* = intraneurite signal fraction; R0 = rotationally invariant signal for L = 0, *b* = 6,000 s/mm^2^; D_eperp_ = extra-axonal perpendicular diffusivity.

Examining a large AOIFCD cohort using ultra strong diffusion gradients, this study identified multiple localized white matter motor pathway differences in the dystonia cohort compared with controls, most notably involving the anterior thalamic radiations, thalamopremotor tracts, and striatopremotor tracts (Figure 6). These differences were not observed when evaluating whole-tract averages (tractography) or during tractometry analysis of other motor tracts (middle cerebellar peduncle, bilateral inferior cerebellar peduncles, superior cerebellar peduncles, corticospinal tracts, superior thalamic radiations, eTable 3) or the optic tracts used as a non-motor comparison. Among the general/nonspecific measures, statistically significant white matter pathway differences were predominantly observed in FA, MK, and RK while measures of orientational dispersion (ODI, *p*_2_) and intra-axonal signal fraction measures (NDI, *f*) were the most discriminative microstructural modeling characteristics.

Differences in AOIFCD seen in the anterior thalamic radiations involved measures associated with lower mid-tract anisotropy (FA), greater orientational dispersion (ODI and *p*_2_), and lower intra-axonal signal fraction (*f*), predominantly involving the left-sided tracts. By contrast, the differences observed in the thalamopremotor tracts involved both mid and distal portions, with higher mid-tract neurite density measures (NDI) and an associated trend toward lower orientational dispersion, compared with lower tract density and higher orientational dispersion in the distal tract portion as it approaches the premotor cortex (ODI, *f*). Corresponding patterns were seen in both subgroups (AOIFCD with and without tremor), and although these did not reach statistical significance in the tremor cohort, this is likely due to the small overall size of this subgroup, which is not adequately powered to confirm small effect sizes. Within the striatopremotor tracts, differences were predominantly seen within the proximal tract where it outflows from the striatum, with lower markers of neurite density (*f*, R0), together with an additional mid-tract difference seen in extra-axonal perpendicular diffusivity, the only difference identified in an extra-axonal marker in this study.

As outlined above, few imaging studies in dystonia have used approaches that assess more specific microstructural features. However, our findings are consistent with a single study in which fixel-based analysis was used to examine a cervical dystonia cohort. In this study, they identified reduced apparent fiber density in the vicinity of the striatum, consistent with our findings of lower neurite density markers in the proximal outflow of the striatopremotor tract.^16^ Our previous work involving NODDI analysis of a mixed dystonia cohort recruited using the UK Biobank identified changes in the superior cerebellar peduncles and distal anterior thalamic radiations in the cervical dystonia cohort. However, in this instance, the work was limited by the level of clinical phenotypic data available with analysis of electronic health records. Despite this, higher thalamopremotor mid-tract FA and regions of lower striatopremotor mid-tract neurite density measures were identified in the dystonia cohort, compared with controls, but their significance did not survive multiple comparison correction.^9^ Both of these previous studies used only moderate *b*-values (maximum *b* = 2,000 s/mm^2^) and a maximum of 2 b-shells, whereas this work used ultra-strong diffusion gradients to perform a multishell acquisition with 8 distinct b-shells up to *b* = 30,000 s/mm^2^, enabling greater accuracy of modeling. The small and localized nature of the identified tract differences indicate that the pathophysiologic changes involved in AOIFCD likely represent subtle, potentially adaptive, microstructural processes, as opposed to widespread pathology. This may be indicative of overutilization of specific motor pathways and underutilization of others, creating imbalance and resulting in unopposed excess muscle activation.

Projections relating to the premotor cortex, linking with both the thalamus and the striatum, have been implicated in this study. This cortical region is considered to play an important role in motor preparation,^39^ with overactivity of the premotor cortex having been implicated in dystonia with both PET and fMRI studies.^1,40-43^ Thalamic projections, modulating activity of the premotor cortex through the thalamopremotor tract, are predominantly excitatory in nature. The findings from this study of higher mid-tract neurite density and lower orientational dispersion potentially indicate overutilization of this portion of the pathway while the changes in the distal tract as it approaches the premotor cortex (greater fiber dispersion) potentially suggest disorganization of this excessive excitatory input. The striatopremotor projections, predominantly providing an excitatory link from the premotor cortex to the striatum, demonstrated differences associated with greater orientational dispersion as the tract approaches the striatum, again potentially indicating disorganization of the excitatory inputs as they approach their target region. Finally, the anterior thalamic radiations largely provide excitatory projections between the prefrontal cortex and thalamus, with the prefrontal cortex involved in higher cortical functions^44^ including executive functioning and other recognized non-motor phenotypes associated with dystonia, such as anxiety and depression.^45,46^ The anterior thalamic radiations demonstrated less cohesive mid-tract orientation, coupled with positive correlation with multiple non-motor symptom severity, predominantly psychiatric and pain-related symptoms (Figure 5). A region in which no differences were observed was the superior cerebellar peduncles, involving the white matter fibers traveling from the cerebellum to the thalami. The cerebellum has been increasingly implicated in dystonia pathogenesis, with postmortem studies demonstrating the presence of torpedo bodies,^47^ animal model studies indicating Purkinje cell ectopic dendritic spines,^48^ and white matter diffusion imaging differences identified in genetic dystonias, with more mixed findings in idiopathic forms.^10^ The absence of statistically significant differences in this cohort may reflect genotypic specific changes in this pathway, rather than a more general feature of dystonia.

The application of acquisitions using an ultra-strong diffusion gradient scanner in this study enables the attainment of higher diffusion weightings per unit time. This enables far greater signal-to-noise ratios than is achievable on an MR scanner with typical gradient strengths. MR scanners used more routinely require lengthier diffusion times to reach the same *b*-values, leading to substantial signal decay with inadequate remaining signal when higher *b*-values are sought. The acquisition here of high *b*-value data spanning multiple b-shells enables greater sensitivity to diffusion properties of tissue. This enables improved tractography because of enhanced ability to determine complex white matter structures, for example, regions of crossing fibers^49^ and better accuracy of microstructural modeling approaches, negating the need for extensive a priori fixing of values, which can lead to model degeneracy.^50^

While these microstructural modeling approaches enable more tissue-feature specific measures, the limitations of these, as with all current microstructural modeling approaches, are that the models are overly simplistic representations of the white matter structure, for example, axons are represented as simple “sticks,” yet comprise subtler shape variations that are not accounted for in current models. In addition, the models view the white matter structure to consist solely of axons, omitting signal contributions from glial cells, cellular projections, and extracellular matrix, the latter having previously been suggested to be of importance in dystonia pathogenesis.^51^ There is likewise a potential contribution to the signal from crossing fibers and the presence of multiple fiber bundles, beyond those corresponding to the tract being measured, which in turn may reduce the sensitivity to tract-specific features. The dominance of between-group differences in areas of higher FA may be reflective of this and may contribute to the difficulty in discerning subtle microstructural localized differences in idiopathic forms of dystonia. This study was also limited by the inability of the method to undertake along-tract profiling of those tracts linking the subcortical and primary sensorimotor cortices, which were, therefore, omitted from analysis, despite being key potential pathways in dystonia pathogenesis. Finally, while analysis of multiple parameters across multiple brain regions can be important in gaining further insights into underlying pathogenesis, this then leads to a requirement for multiple comparison corrections, which in themselves can result in false-positive and negative findings being identified.

The analysis identified substantial correlations between a multitude of non-motor measures and the significant tract regions; while this may indicate that the non-motor phenotype is closely linked with the observed microstructural tissue features, it must be noted that these correlations were a secondary analysis and not the primary goal of the study. Further dedicated work is needed to determine whether these MR measures are robustly correlated with phenotype in a clinically meaningful way. Finally, not assessed here is whether phenotypes with evidence of higher correlations would apply independent of disease status and whether they account for any of the between-group variability observed in these findings.

Overall, this work identifies localized changes along white matter motor pathways in a deeply phenotyped cohort of patients diagnosed with AOIFCD, compared with controls. White matter projections linking to the premotor and prefrontal cortices seem to be predominantly implicated, with differences in AOIFCD appearing to predominantly affect the dispersion of fiber orientation and intra-axonal compartment measures. Further work is needed to replicate these findings in larger cohorts and coupled with the need for histopathologic correlation, to gain greater confidence of the underlying structural changes that give rise to the observed imaging differences.

## Appendix Authors

**Table:**

Name	Location	Contribution
Claire L. MacIver, MBBCh, BSc, MSc	Cardiff University Brain Research Imaging Centre, Cardiff University; Neuroscience and Mental Health Research Institute, Division of Psychological Medicine and Clinical Neurosciences, Cardiff University School of Medicine, United Kingdom	Drafting/revision of the manuscript for content, including medical writing for content; major role in the acquisition of data; study concept or design; analysis or interpretation of data
Derek Jones, PhD	Cardiff University Brain Research Imaging Centre, Cardiff University, United Kingdom	Drafting/revision of the manuscript for content, including medical writing for content; study concept or design
Katy Green	Cardiff University Brain Research Imaging Centre, Cardiff University, United Kingdom	Major role in the acquisition of data
Konrad Szewczyk-Krolikowski, DPhil	North Bristol NHS Trust, United Kingdom	Major role in the acquisition of data
Andre Doring, PhD	Cardiff University Brain Research Imaging Centre, Cardiff University, United Kingdom	Major role in the acquisition of data
Chantal M.W. Tax, PhD	Cardiff University Brain Research Imaging Centre, Cardiff University, United Kingdom; Image Sciences Institute, University Medical Center Utrecht, the Netherlands	Drafting/revision of the manuscript for content, including medical writing for content; study concept or design; analysis or interpretation of data
Kathryn J. Peall, MD, PhD, FRCP	Neuroscience and Mental Health Research Institute, Division of Psychological Medicine and Clinical Neurosciences, Cardiff University School of Medicine, United Kingdom	Drafting/revision of the manuscript for content, including medical writing for content; study concept or design; analysis or interpretation of data

## Glossary

AK: axial kurtosis
AOIFCD: adult-onset idiopathic focal cervical dystonia
BFMDRS: Burke-Fahn-Marsden Dystonia Scale
CANTAB: Cambridge Neuropsychological Test Automated Battery
DKI: diffusion kurtosis imaging
DTI: diffusion tensor imaging
FA: fractional anisotropy
FSL: FMRIB Software Library
MD: mean diffusivity
MK: mean kurtosis
NDI: Neurite Density Index
NODDI: neurite orientation dispersion and density imaging
ODI: Orientation Dispersion Index
RK: radial kurtosis
SF36: Short Form 36 Health Survey
SM: standard model
TWSTRS: Toronto West Spasmodic Torticollis Rating Scale
